# An Improved Genetic Algorithm for the Recovery System of USVs Based on Stern Ramp Considering the Influence of Currents

**DOI:** 10.3390/s23198075

**Published:** 2023-09-25

**Authors:** Lulu Zhou, Xiaoming Ye, Zehao Huang, Pengzhan Xie, Zhenguo Song, Yanjia Tong

**Affiliations:** 1 School of Energy and Power Engineering, Huazhong University of Science and Technology, Wuhan 430074, China; zhoululu1225@163.com (L.Z.); xpz564941004@163.com (P.X.); u202010679@hust.edu.cn (Y.T.); 2 China Ship Development and Design Center, Wuhan 430064, China; howardze@163.com (Z.H.); song850624@126.com (Z.S.)

**Keywords:** the unmanned surface vehicle, path planning, genetic algorithm, line of sight method, autonomous guidance and recovery, stern ramp recovery

## Abstract

With the progression of marine exploration and exploitation, as well as the advancements in mechanical intelligence, the utilization of the unmanned surface vehicle (USV) and the design of their guidance system have become prominent areas of focus. However, the stern ramp recovery of the USV is still in its infancy due to its unique attitude requirements and automation design. Furthermore, few studies have addressed the impact of maritime disturbances, with most research limited to simulations. To enhance the efficiency and accuracy of stern ramp recovery, this paper presents the development and construction of a novel recovery system. By incorporating physical modeling of disturbance forces acting on USVs at sea, the practicality of the system is improved. Additionally, an optimized genetic algorithm is introduced in the navigation module to improve convergence rates and subsequently enhance recovery efficiency. A line-of-sight (LOS) algorithm based on average velocity is proposed in this paper to ensure the attainment of unique attitude requirements and to improve the effectiveness of stern chute recovery. This paper provides a detailed description of the independently designed USV hardware system. Moreover, simulations and practical experiments conducted using this experimental platform are presented, offering a new solution for the USV’s stern ramp recovery.

## 1. Introduction

If you ask chatGPT how much of the ocean has yet to be explored by mankind, it will tell you that over 80% of the ocean remains unmapped, unobserved, and unexplored. To be honest, we have no idea exactly how much of the ocean is uncharted by humanity. But we cannot deny that there has been an increasing enthusiasm and confidence among researchers in the development of ocean exploration technologies in recent years. Driven by oceanographic research and other marine equipment requirements, the unmanned surface vehicle (USV) has gained worldwide attention for its remarkable autonomy and mission assistance capabilities.

**Figure 1:**
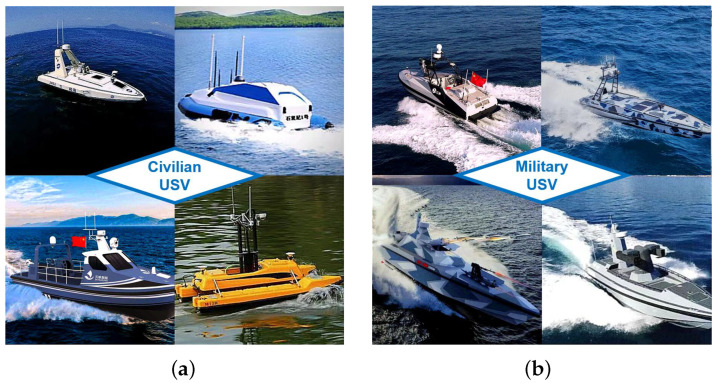
(**a**) Unmanned Surface Vehicle (USV) for civilian use; (**b**) USV for military use [5,6,7,8,9,10,11,12].

Compared to other vessels, the USV has the advantage of being smaller and operating more agilely, requiring only sufficient space for the relevant sensors and auxiliary navigation devices to make sure the vehicle functions properly. Moreover, the USV can be applied in extreme conditions independently, such as strong waves, tides, and radiation leaks, by means of a predefined system or remote control by professionals; thus, the safety of the operators can be ensured. Due to a variety of these aforementioned advantages, the USV has been well-accepted in both civilian and military fields in recent years [1,2,3,4], as shown in Figure 1.

**Figure 2:**
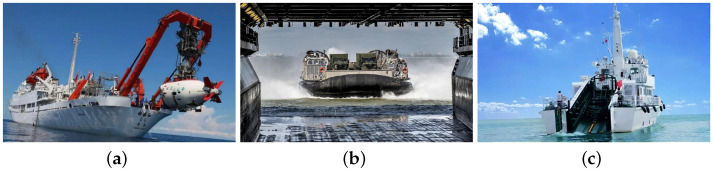
(**a**) Hoist-based recovery requires the installation of davits, parking racks, and towing devices on board the mother ship. To complete the unhooking operation, manual assistance is needed [13]. (**b**) The recovery enables small boat stowage on racks in a well-deck, and once the water reaches a certain depth, the release mechanism activates for deployment [14]. (**c**) The stern ramp of a ship [15].

Unlike maritime navigation, the recovery situation of the USV does not need to consider the International Regulations for Preventing Collisions at Sea (COLREGS) for its immediate environment, close to the stern ramp of its large target ship, without the interference of other vessels. The intelligence of the stowage and release process of the USV will have a direct impact on the efficiency of its mission performance. It is mainly divided into hoist-based recovery, well-deck recovery, and stern ramp recovery. Examples are shown in Figure 2. The hoist-based recovery method shows advanced development with minimal modifications to the mother ship’s hull, but recovery speed is highly affected by water surface conditions, leading to challenging bracket alignment and extended retrieval times. In contrast, the well-deck recovery technique accommodates complex water surface conditions and achieves rapid retrieval speeds but requires greater structural demands on the mother ship and the inclusion of a spacious docking bay.

The stern ramp recovery technology exhibits superior overall performance in terms of the mother ship’s hull structure requirements, adaptability to water surface conditions, and stowage efficiency, making it highly suitable for the rapid autonomous recovery of USVs. This method not only allows the mother ship to retract and release small boats at higher speeds, but also adapts to sea conditions of level 6. This advantage allows the mother ship to choose the appropriate timing for flushing. The primary operating principle of the stern ramp recovery system is that during recovery, the USV navigates a predetermined trajectory toward the mother ship’s aft section. During this approach, the USV continuously adjusts its heading angle to ensure alignment with the centerline of the mother ship’s stern ramp. In addition, it maintains a specified distance from the aft section of the mother ship while staying on course. However, achieving precise centering and control near the stern ramp remains challenging. The low dependency on manual assistance from the mothership during the recycling process results in high autonomous navigation accuracy and strong resistance to wake interference from the USV. This places higher demands on the convergence rate of the USV path planning algorithm and the accuracy of the recovery system. The development of autonomous recovery technology for USVs is still in its nascent stages.

Due to the special requirements of stern ramp recovery for the path and USV’s attitude, the question of which path planning algorithm to choose is particularly important for the establishment of the recovery system. We hope that factors such as resistance to ocean current disturbance, smooth motion, accurate heading angle, and reasonable dynamic parameters of the USV are comprehensively considered in the proposed recycling system. Therefore, the algorithm used should have a multidimensional evaluation system rather than a single optimization method. In comparison with other intelligent algorithms, the genetic algorithm (GA) has significant advantages due to its adaptive evaluation indicators. Also, the genetic algorithm does not fall into the local optimum problem as easily as gradient-based optimization algorithms. For this reason, we chose this algorithm and optimized it to be combined with the recycling system. Furthermore, a suitable control system for the dynamics of the USV is imperative from a hardware perspective. To enhance the realism of the simulation in a maritime environment, we also conducted modeling of the interference environment and incorporated it into the tracking algorithm. Our main contributions are summarized as follows.

- We conducted physical modeling of currents and waves to analyze the impact of USV movement in a water environment from a force perspective. Subsequently, we applied these findings to our tracking algorithm in order to enhance the accuracy and effectiveness of the tracking process.
- To meet the specific requirements of stern ramp recovery, we enhanced the genetic algorithm by incorporating three-dimensional modeling and genetic manipulation techniques. This resulted in an improved convergence rate of the algorithm. Additionally, we designed a multi-dimensional evaluation index fitness function to streamline the screening mechanism.
- We improved the tracking algorithm and introduced a new tracking algorithm specifically designed for average speed based on line of sight (LOS). This exclusive algorithm ensures the effectiveness of waypoint tracking.
- We independently constructed the experimental platform and devised a hardware connection system within the USV. This system serves as a solution for implementing the algorithm in the actual operation of the USV.

## 2. Related Works

Based on the detailed introduction of the recycling method provided in the previous section, this section will primarily focus on presenting the relevant methods employed by scholars for unmanned boat path planning. Furthermore, we will elucidate the rationale behind our selection of the genetic algorithm and LOS as the research methodology.

### 2.1. The Planning Algorithm

Currently, path planning algorithms used for USVs in domestic and international research can be classified into four main categories: graph search algorithms, virtual potential field methods, random sampling algorithms, and intelligent algorithms.

Graph search algorithms are considered classical path planning algorithms; however, their usage has declined in recent years due to the slow computational speed of individual underlying logic. Among these algorithms, the A* algorithm has garnered attention for its remarkable scalability. Singh et al. [16] considered moving ships as quasi-static entities and other ships as static obstacles during the current planning time for map modeling. They used the A* algorithm to search for collision avoidance paths by incorporating the notion of ship safety. Simulation results demonstrated the effectiveness of the algorithm in effectively avoiding moving ships while achieving commendable real-time performance.

The basic concept of the virtual potential field (VPF) method is to construct a virtual potential field within the map using certain techniques. The path is then generated using the gradient descent algorithm. However, this method suffers from inherent limitations such as local minima and oscillations. Kim et al. [17] integrated the method with the velocity obstacle method by incorporating a repulsive force field associated with the encountered velocity obstacle between two ships into the classical potential field. This fusion approach enabled dynamic collision avoidance for USVs. Similarly, Sang et al. [18] used a hierarchical programming approach. They first used the A* algorithm to generate an initial path and then used an improved artificial potential field (APF) method to generate the desired USV formation.

Random sampling methods exhibit varying convergence rates, making them extensively employed in the context of Multi-Agent Path Finding (MAPF) due to their inherent efficacy. Lee et al. [19] introduced a novel approach called the grafting RRT algorithm to achieve dynamic collision avoidance in USVs which uses the speed obstacle method to detect potential collisions and calculates the grafting angle. By generating and inserting grafting points based on this angle, successful dynamic collision avoidance is achieved.

In terms of obstacle avoidance, Khan et al. introduce a penalty term in the objective function, so that the tracking control and obstacle avoidance problems are unified into a constrained optimization problem, and active rewards are given to obstacle evaders [20]. As objectives become more complex, intelligent algorithms, such as the genetic algorithm, excel in path planning. It utilizes genetic manipulations like mutation and crossover to enhance diversity in the sample population, enabling efficient multi-target search. However, the traditional genetic algorithm exhibits low convergence rates in USV path planning and heavily relies on well-designed genetic operators. To address these limitations, Wang et al. [21] propose a combination of GA and fuzzy APF for hierarchical path planning, effectively adapting to unpredictable environments. Nonetheless, challenges persist in handling time-varying dynamic obstacles. Xin et al. [22] suggest mitigating these issues by increasing the number of superior offspring through multi-domain inversion and second fitness evaluation. Nevertheless, this approach introduces additional complexities to the algorithm and encoding process.

### 2.2. The Tracking Algorithm

The purpose of tracking control is to manipulate the propulsion system, such as the propeller and rudder, based on a rational tracking control law. This enables the USV to navigate along the intended trajectory determined by the path planning module. Depending on whether the trajectory includes a time dimension, the problem can be divided into two categories: path tracking and trajectory tracking. The stern ramp recovery involves dynamic obstacle avoidance and requires a higher speed than the mother ship during the slope flushing phase. Therefore, it is a trajectory tracking problem.

Pettersen [23] used the LOS algorithm to compute the desired heading angle and combined it with a cascaded feedback controller to control the yaw torque. This approach achieved linear tracking of the USV at a constant velocity. Fossen [24], using kinematic models for both USV and Unmanned Aerial Vehicle (UAV), rigorously proved the unified semi-global exponential stability of path tracking under LOS control. This contribution enriched the theoretical foundation of the LOS guidance law.

To improve the tracking stability and accuracy of the USV under environmental disturbances such as wind, waves, and currents, Caharija [25] introduced an integral term into the classical LOS guidance. This term compensated for the lateral drift of the USV and mitigated tracking biases caused by environmental disturbances. Building on this work, Fossen [26] used a nonlinear adaptive controller to achieve two-dimensional Dubins curve path tracking. Simulation results demonstrated the successful application of this tracking control method in accurately tracking the USV even under significant drift angles induced by wind, waves, and power disturbances.

Liu [27] proposed an improved LOS tracking algorithm based on prediction. This algorithm was designed for tracking underactuated USVs, effectively compensating the uncontrollable sideslip caused by marine environmental disturbances through adaptive terms. In addition, this study cascaded the tracking error system with the prediction error system and rigorously demonstrated the consistent global stability of the entire system.

## 3. Recovery System of USVs Based on Stern Ramp

**Figure 3:**
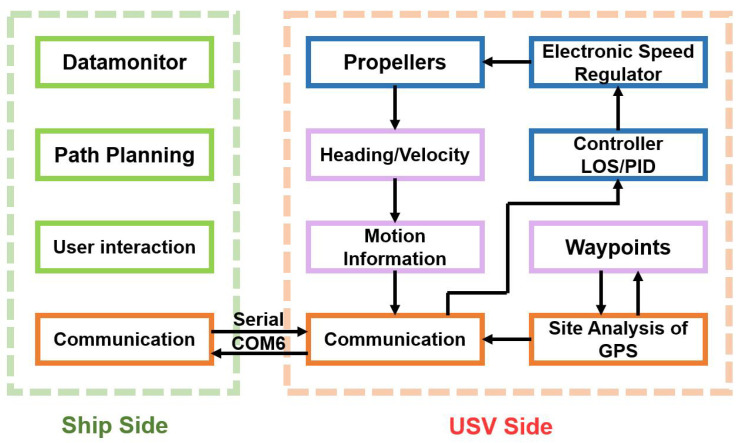
The recovery system structure of our USV.

Due to the particular environment of the autonomous recovery using a stern ramp, most of the recovery strategies have less concern about interference caused by currents as well as the effects of malposition on recovery effects. For a successful ramp approach, it is essential to have sufficient power and to approach the ramp at an optimal angle. This combination guarantees a seamless and proficient transition onto the ramp surface, facilitating a smooth and efficient docking process. To ensure the safety and efficiency of the recovery process, we have developed an innovative stern ramp recovery system that prioritizes three key factors: precise visibility, seamless guidance, and stable navigation. The recovery system structure is shown in Figure 3.

Enter the initial parameters for the planning algorithm into the upper computer, wait for it to complete the calculations, and then package and transmit the set of waypoint information generated by the genetic algorithm to the USV end. This set of information includes the coordinates, real-time velocities, and headings of each waypoint. Upon receiving the information, the PID controller actuates the electronic speed regulators to regulate the propellers, thereby adjusting the speed and heading of the USV. Meanwhile, the LOS controller accurately tracks the waypoint to prevent interference such as currents from causing the USV to deviate from the planned route. In the meantime, the actual position information of the USV obtained by the Global Positioning System (GPS) will also be sent to the upper computer together with the current speed and the heading.

### 3.1. Precise Visibility Establishment of a Current Interference Model for Unmanned Surface Vehicles

The recovery process of the USV is affected by external environmental disturbances, which have a significant impact on the speed and stability of the centering procedure. Therefore, it is essential to develop a suitable mathematical model to account for these external disturbances. Factors such as sea currents and waves particularly affect the autonomous guided recovery motion.

**Figure 4:**
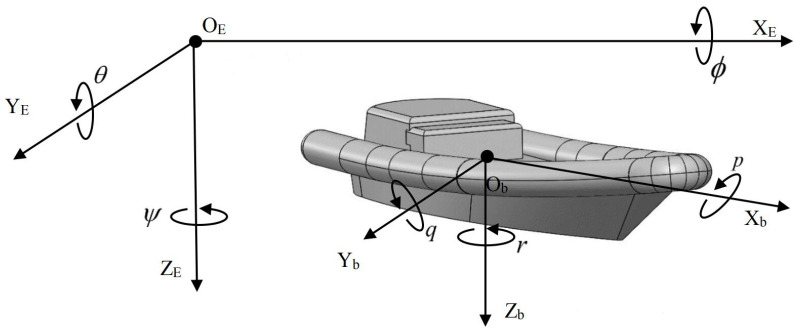
The geodetic coordinate system and the follower coordinate system.

The motion of the USV can be summarized into 6° of freedom, and in order to accurately describe the motion state of the USV, a combination of a geodetic coordinate system and follower coordinate system is used to describe the position and attitude information, as shown in Figure 4.

Assuming that the center of gravity of the USV aligns with the center of the follower coordinate system, we can deduce the equation of motion governing the forces exerted on the USV during its movement. This equation is derived from the Newton–Euler equations for rigid body motion in a fluid medium and is given as Equation (1).

(1) $$\left\{\begin{array}{c} m\dot{u}-mvr=F_{x}\\ m\dot{v}-mvr=F_{y}\\ I_{G}\dot{r}=F_{n} \end{array}\right.$$

where *m* denotes the quality of the USV, *v* and *u* present the transverse and longitudinal velocities, *r* is the bow-swinging angular velocity, $I_{G}$ is the moment of inertia of the USV on the z-axis, and $F_{x}$, $F_{y}$, and $F_{n}$ are the force in the x-direction and y-direction and the moment.

#### 3.1.1. Current Interference Model

The disturbance force and disturbance moment experienced by the USV due to sea currents can be attributed to two main components: (1) viscous resistance, arising from the friction and pressure difference between the USV and the surrounding fluid; (2) inertial resistance, resulting from the circulation patterns near the USV and the influence of the free liquid surface. The equations are given by Equation (1).

(2) $$\left\{\begin{array}{c} F_{x}^{current}=\frac{1}{2}\rho S_{f}V_{c}^{2}C_{x}\left(\beta \right)\\ F_{y}^{current}=\frac{1}{2}\rho S_{r}V_{c}^{2}C_{y}\left(\beta \right)\\ F_{n}^{current}=\frac{1}{2}\rho S_{r}V_{c}^{2}C_{n}\left(\beta \right) \end{array}\right.$$

where $V_{c}$ is the flow velocity; $S_{f}$ is the forward projected area of the USV above the waterline; $S_{r}$ is the side projected area of the USV above the waterline; β is the encounter angle; $C_{x}\left(\beta \right)$, $C_{y}\left(\beta \right)$, $C_{n}\left(\beta \right)$ are the test coefficients.

#### 3.1.2. Wave Interference Model

The wave force can be categorized into two components: the first-order wave force and the second-order wave force. The former induces vertical oscillation of the USV with a fixed period, without altering its total mechanical energy. On the other hand, the latter causes the USV to experience time-varying horizontal thrust, resulting in horizontal displacement [28], which influences the autonomous guided recovery process. Hence, we disregard the impact of the first-order wave force and focus our analysis on the effect of the second-order wave force on the USV during its autonomous guided recovery.

During the recovery process, the wave force primarily originates from the wind and the wake of the mother ship. The drift force model proposed by Daidola [29] enables us to derive the wave force from the wave model, as depicted in Equation (3):

(3) $$\left\{\begin{array}{c} F_{x}^{wave}=\frac{1}{2}\rho L\xi^{2}\cos\chi W_{x}\left(\lambda_{w}\right)\\ F_{y}^{wave}=\frac{1}{2}\rho L\xi^{2}\sin\chi W_{y}\left(\lambda_{w}\right)\\ F_{n}^{wave}=\frac{1}{2}\rho L\xi^{2}\sin\chi W_{n}\left(\lambda_{w}\right) \end{array}\right.$$

where ξ is the average wave amplitude; χ is the encounter angle; $\lambda_{w}$ is the wave wavelength; $W_{x}\left(\lambda_{w}\right)$ and $W_{y}\left(\lambda_{w}\right)$ are the wave drift coefficients; $W_{n}\left(\lambda_{w}\right)$ is the drift moment coefficient around the z-axis. The specific calculations are shown in Equation (4).

(4) $$\left\{\begin{array}{c} \xi =h_{w}/2\\ W_{x}\left(\lambda_{w}\right)=0.05-0.2\left(\frac{\lambda_{w}}{L}\right)+0.75{\left(\frac{\lambda_{w}}{L}\right)}^{2}-0.51{\left(\frac{\lambda_{w}}{L}\right)}^{3}\\ W_{y}\left(\lambda_{w}\right)=0.46+6.83\left(\frac{\lambda_{w}}{L}\right)-15.65{\left(\frac{\lambda_{w}}{L}\right)}^{2}+8.44{\left(\frac{\lambda_{w}}{L}\right)}^{3}\\ W_{n}\left(\lambda_{w}\right)=-0.11+0.68\left(\frac{\lambda_{w}}{L}\right)-0.79{\left(\frac{\lambda_{w}}{L}\right)}^{2}+0.21{\left(\frac{\lambda_{w}}{L}\right)}^{3} \end{array}\right.$$

where $h_{w}$ is the wave height. The relationship between the height of the wave $h_{w}$, the wave period $T_{wave}$, the wavelength $\lambda_{w}$, and the wind speed $V_{R}$ is shown in Equation (5).

(5) $$\left\{\begin{array}{c} h_{w}=0.015V_{R}^{2}+1.5\\ T_{wave}=-0.001V_{R}^{3}+0.042V_{R}^{2}+5.6\\ \lambda_{w}=0.54T_{wave}^{2} \end{array}\right.$$

### 3.2. Seamless Guidance Path Planning Algorithm for Stern Ramp Recovery

The quality, efficiency, and convergence of path planning algorithms are crucial factors to consider. The genetic algorithm, known for its intelligent nature and multi-evaluation index mechanism, offers significant advantages in global path planning. However, traditional genetic algorithms rely on two-dimensional raster graphs, which suffer from time-consuming computations, unstable algorithmic architectures, and non-uniqueness in convergence rate performance. To address these limitations, we propose a novel approach that combines the characteristics and requirements of stern chute recovery, focusing on optimizing waypoint distribution, improving population initialization, and utilizing advanced genetic methods to establish a more suitable path planning algorithm.

#### 3.2.1. Novel Collision Avoidance Based on Three-Dimensional Modeling

Considering the wide range of actual distances in latitude and longitude recorded by GPS, it becomes necessary to perform additional processing on the coordinates. In our simulation process and program design, we use the following coordinate information transformation formula for actuation:

(6) $$\left\{\begin{array}{c} X_{USV}=\left(X_{GPS}-X_{0}\right)/100,000\\ Y_{USV}=\left(Y_{GPS}-Y_{0}\right)/100,000 \end{array}\right.$$

where $X_{USV}$ and $Y_{USV}$ denote the current location of the USV, $X_{GPS}$ and $Y_{GPS}$ present the information GPS received, and $X_{0}$ as well as $Y_{0}$ indicate the position receiver placed.

It should be noted that there is a trade-off between the computational time and the resolution [30]; if the resolution is higher, a longer computational time will be required. However, while streamlining environment modeling can improve algorithm efficiency and convergence rate, as well as reduce spatiotemporal complexity, it also introduces potential drawbacks. These include sharper corners and excessively long sections in the planned path, resulting in rougher paths that hinder smooth evolution. Therefore, it is crucial to establish a robust algorithmic operation mechanism based on a well-designed pre-processing method for the modeling environment. This ensures the practical feasibility of the planning path.

**Figure 5:**
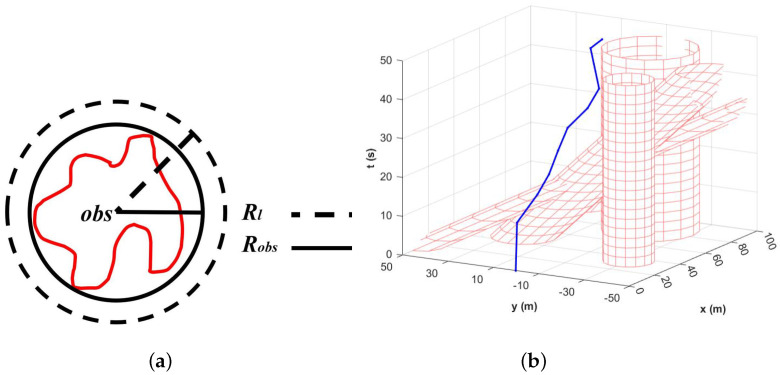
(**a**) The circular bounding boxes. (**b**) The planning path in 3D coordinate system. The blue line presents the planning path.

Considering the sparsely distributed obstacles on the water surface, we have opted to use circular bounding boxes to represent these obstacles, as illustrated in Figure 5a. The outer circle of the actual obstacle boundary is used as the obstacle in the planning space, aligning with the cornering motion dynamics of the USV in a water environment. This approach significantly reduces memory consumption associated with environment modeling, as it only requires storing a few essential data points such as center coordinates, radius, and movement speed for each obstacle. Taking into account the shifting caused by interference and the error of being labeled as a particle, we add the compensation radius to the boxes. The equation is given by Equation (7).

(7) $$R_{l}=R_{obs}+D_{USV}+R_{c}$$

where $R_{l}$ denotes the minimum radius of collision avoidance distance from the USV to the center of the circular bounding box, $R_{obs}$ represents the initial radius of the box, and the whole compensation radius is the length of the hull $D_{USV}$ plus the possible displacement $R_{c}$ caused by environmental interference, which is usually taken as 1 m.

Upon introducing the time dimension into the modeling, the bounding boxes of the obstacles are represented as oblique or straight cylinders in the x-y-t coordinate system. The expression of the three-dimensional geometry is depicted in Equation (8).

(8) $$\left\{\begin{array}{c} {\left(X-X_{obs}+V_{x}t\right)}^{2}+{\left(Y-Y_{obs}+V_{y}t\right)}^{2}=R_{obs}^{2}\\ V_{x}=0\;m/s\;when\;static\;obstacle\\ V_{y}=0\;m/s\;when\;static\;obstacle \end{array}\right.$$

where $X_{obs}$ and $Y_{obs}$ denote the abscissa and ordinate of the center of the circular bounding boxes, $R_{obs}$ represents its radius, and $V_{x}$ and $V_{y}$ denote the velocity in the x-direction and velocity in the y-direction.

In order to simplify the mathematical modeling of obstacle avoidance, we made the following assumptions.

- Dynamic obstacles move in uniform linear motion.
- The USV performs segmented uniform acceleration linear motion, with each consecutive pair of waypoints forming a segment.

By solving for temporal intersections between the motion trajectory of the USV and the movement trajectory of the obstacles, we can determine whether the proposed planning will result in a collision. The relevant equation is given by Equation (9).    

(9) $$\begin{array}{c} \left[{\left(\bar{V_{Uy}}+V_{Oy}\right)}^{2}+{\left(\bar{V_{Ux}}+V_{Ox}\right)}^{2}\right]T^{2}\\ +2\left[\left(\bar{V_{Uy}}+V_{Oy}\right)\left(Y_{usv}-Y_{obs}\right)+\left(\bar{V_{Ux}}+V_{Ox}\right)\left(X_{usv}-X_{obs}\right)\right]T\\ +\left[{\left(X_{usv}-X_{obs}\right)}^{2}+{\left(Y_{usv}-Y_{obs}\right)}^{2}-R_{obs}^{2}\right]=0 \end{array}$$

where $\bar{V_{Ux}}$ and $\bar{V_{Uy}}$ are the average velocities of the certain segment, and $V_{Ox}$ and $V_{Oy}$ are the velocities of the obstacle bounding boxes.

Given the particularity of stern ramp recovery, we adopt the horizontal line representing the stern ramp as the x-axis in a 2D plane. This division separates the water into two symmetrical parts, one above and one below the stern ramp. The distribution of obstacles and the performance of the planned path are shown in Figure 5b.

#### 3.2.2. Improvements in Genetic Manipulation

In this paper, the genetic algorithm selects the population as the solution set for the recovery paths. Each path is represented by a distinct genetic individual. These individuals encompass three-dimensional coordinate information of multiple waypoints, storing the X, Y, and T coordinates along with real-time speed. The enhanced global planning algorithm autonomously determines the number of gene loci based on the input start and end points. This effectively enhances the computational capability of the genetic algorithm. Rather than generating the waypoints randomly, we designate the X-axis direction as the step direction and establish the range of intervals for each waypoint based on the step size $D_{SL}$. The length of $D_{SL}$ varies according to different scenarios and accuracy requirements.

**Figure 6:**
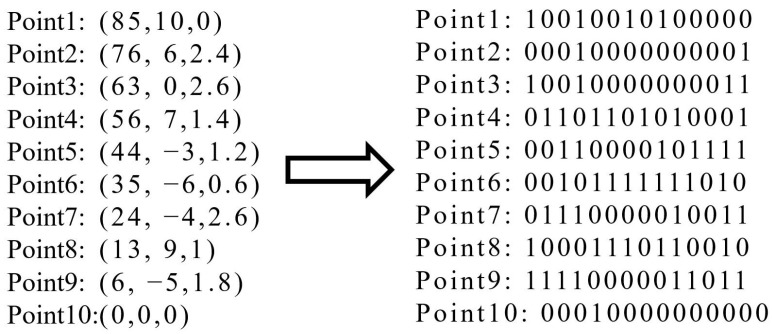
The translation process of the encoding.

After that, the waypoints will then be generated in each interval in turn. The code conversion between binary and decimal is shown below.

(10) $$\left\{\begin{array}{c} X_{tr}=X_{i}-D_{SL}\cdot \left(i-1\right)-1\\ Y_{tr}=Y_{i}+64\\ V_{tr}=V_{i}\cdot 5 \end{array}\right.$$

where $X_{tr}$ and $X_{i}$ denote the abscissa of binary system and decimal system. Ordinate and velocity are presented in a similar way.

(11) $$\left\{\begin{array}{c} X_{i}=\sum_{n=1}^{3}m\cdot 2^{\left(n-1\right)}+1\\ Y_{i}=\sum_{n=4}^{10}m\cdot 2^{\left(n-1\right)}-64\\ V_{i}=\sum_{n=11}^{14}\frac{m\cdot 2^{\left(n-1\right)}}{5}\\ T_{i}=\sum_{n=1}^{N-1}\frac{\sqrt{{\left(X_{n+1}-X_{n}\right)}^{2}+{\left(Y_{n+1}-Y_{n}\right)}^{2}}\cdot 2}{V_{i}+V_{i+1}} \end{array}\right.$$

where *n* represents the number of bits in the binary sequence and *N* represents the total number of waypoints. An example of the encoding is shown in Figure 6.

The binary sequence serves as the fundamental basis for genetic algorithm mutation. However, a common issue arises with value overflow subsequent to decoding. Consequently, a mapping procedure is introduced to the binary sequence, involving binary conversion, decoding, and subsequent restoration of the original decimal sequence through inverse mapping. This measure effectively addresses the challenge of overflow encountered in the process.

**Figure 7:**
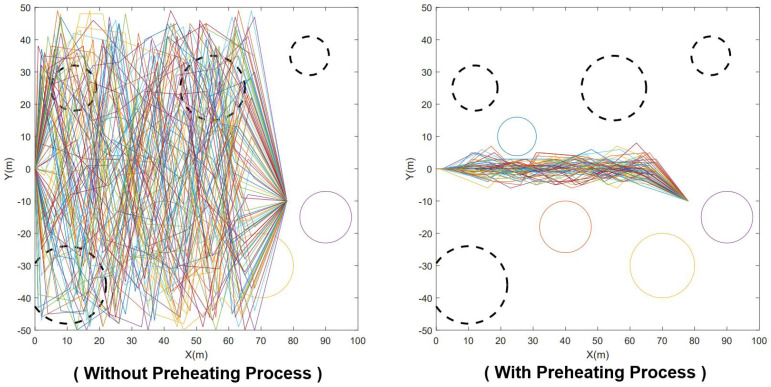
The comparison on whether to adopt the preheating process during population initialization. The results were simulated with the assistance of MATLAB R2018a (MathWorks, Natick, MA, USA), taking the starting point of (78, −10) as an example. In this picture, the dashed circles present dynamic obstacles.

The collision avoidance operations of the traditional genetic algorithm, based on 2D raster maps, consume significant memory resources and require additional time. To address this issue, as mentioned in the previous section, we employed a memory-efficient obstacle design during the 3D modeling process. This approach enables collision avoidance operations during population initialization. As illustrated in Figure 7, this preheating process facilitates the generation of high-quality initial individuals and enhances the convergence rate of the algorithm.

The traditional genetic algorithm does not achieve complete global convergence. During the genetic variation process, each individual has the possibility of undergoing crossover and mutation, resulting in changes in their sequence and original expression type. This mechanism serves as the primary means for the genetic algorithm to converge. However, this approach may cause individuals with excellent phenotypes to lose their advantageous genes, thereby reducing the retrieval speed [31].

Many scholars have demonstrated that the genetic algorithm with Elitism Strategy achieves global convergence [32]. After population initialization, the fitness function of genetic individuals is evaluated, and the superior individuals are preserved in the elite group. This prevents the alteration of advantageous phenotypes during the selection process. In each generation, the individuals in the elite group will be replicated to the next generation, continuously updating the set [33]. Typically, around 10% of the elite sequences are retained. Incorporating this method helps improve the convergence speed of the genetic algorithm and reduces the number of iterations.

**Figure 8:**
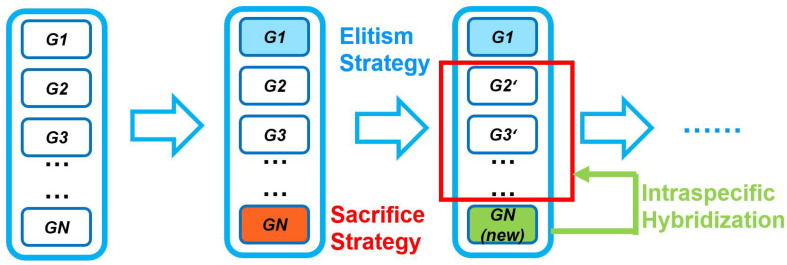
The novel genetic manipulation.

Due to the requirements of rollover prevention caused by inappropriate heading and large turns in the surface environment, we add dynamic metrics to the fitness function to evaluate the performance of USVs. In cases where the generated path does not satisfy the constraints of obstacle avoidance and dynamic metrics, the fitness function is assigned a value of 0. By implementing the Elitism Strategy, individuals with superior genetic traits are preserved, thus minimizing the impact of discarding individuals with undesirable traits on the overall population. In addition, the presence of defective individuals during the evolutionary process consumes computational memory resources, resulting in reduced computational efficiency and increased traversal time of the algorithm [34]. To address this issue, this study proposes a novel strategy called the Strategy of Sacrifice and Intraspecific Hybridization (SSIH), as shown in Figure 8. This strategy emulates natural processes observed in biological populations, ensuring both the feasibility of the generated planning path and improving the convergence rate.

Individuals with a fitness function of 0 are treated as individuals that have died after the population iteration, and their memory is released when the algorithm is implemented. Subsequently, a local initialization scheme is employed to allocate a new path planning to the discarded genetic individuals, considering them as newly generated offspring within the population. In the subsequent genetic mutation operation, these regenerated offspring are hybridized with other genes present in the population. Simulation experiments demonstrate that this operation reduces the convergence time by approximately 10 s and minimizes the number of iterations required, thereby endowing the genetic algorithm with a notable real-time advantage in dynamic obstacle avoidance.

The genetic algorithm is designed to seek optimal solution outcomes. In the event that the quality of the generated path fails to meet the threshold established by the program, the iteration process will persist. In order to uphold real-time planning efficacy, we have imposed a maximum iteration limit of 500. Through extensive simulations and empirical observations, we have determined that this value significantly surpasses the number of iterations necessary to yield results that align with the desired objectives. The pseudo code is shown in Algorithm 1.

Using an ex ante method to analyze, the computational complexity of the proposed genetic algorithm is $O(N^{2})$. But the embedded do-while loop is used for population initialization, which has a very small computational footprint. In practical recovery applications, the execution time of the algorithm is about 2 s and the relevant results will be discussed in Section 2.

#### 3.2.3. New Evaluation Mechanism for the Fitness Function

The implementation of a fitness function aims to enhance the initially subtle distinctions among individuals in the population through calibration and dynamic mapping. This process facilitates the clear manifestation of the variations in phenotypic excellence between different individuals and enables a more effective selection of genetically qualified individuals.

However, while penalty functions can be applied to constraints of any type (linear or nonlinear), their performance is not always satisfactory. Achieving constraint optimality through penalty functions relies on the discovery of appropriate penalty parameters. Even so, determining these parameters often necessitates numerous experiments and has posed a challenge for researchers [35]. Khan et al. proposes an algorithm that uses nature-inspired optimization methods in 2020 to directly solve nonlinear optimization problems without the need for any transformations in robot tracking methods [36].
**Algorithm 1** Optimized Genetic Algorithm**Input:** 
$\left(X_{0},Y_{0}\right)$: start point; width,length:the size of map; $\left(X_{USV},Y_{USV},V_{USV}\right)$: the current information of the USV;
**Output:** getPath(): the planning path which consists of waypoints;
1:set $D_{L}=D_{best}$, set popsize=Num, $Fitness_{value}=F_{Max}$, Nmax;2:Foundation(); //generate initial population3:m=0, k=0;4:**while** m<popsize; **do**5:    i=1, n=0;6:    **while** i<genePoint; **do**7:        **if** isCollision($Point_{i}$,$Point_{i-1}$) == False **then**8:           put $Point_{i}$ into the geneList;9:        **else**10:           restart point generating;11:        **end if**12:        i++, n++;13:        **if** n>Nmax **then**14:           break; //setting a threshold to prevent program crashes15:        **end if**16:    **end while**17:    m++;18:**end while**19:Fitness_calculating(); //get the $Fitness_{max}$;20:**while** $Fitness_{max}<Fitness_{value}$ **do**21:    Elit_Strategy(); //implement the Elit Strategy22:    Tournament(); //select a portion of individuals with strong adaptability23:    TransferBinary(); //binary encoding conversion24:    Variation_binary(); //genetic variation25:    TransferDecimal(); //decimal encoding conversion26:    TimeReset();27:    FitnessReset(); //recalculate fitness function28:    Intraspecific();29:    k++;30:    **if** k>=500 **then**31:        break; //set maximum convergence generation to prevent too much time cost32:    **end if**33:**end while**34:getPath();


We propose a set of adaptation functions that incorporate a penalty weight allocation mechanism. A novel nonlinear fitness relation is established by penalizing the total distance, time spent, degree of alignment, and amount of cornering in recovery planning. In addition, this approach combines the constraints related to collision avoidance with those concerning the dynamics of the USV. While we currently cannot overcome this limitation, our approach simplifies this process by introducing various indicators.

Quantitative indicators and fitness can be calculated by the following equation:

(12) $$\left\{\begin{array}{c} Fitness\left(x\right)=G_{acc}G_{vel}G_{col}\frac{A}{a\cdot Fit_{d}+b\cdot Fit_{T}+c\cdot Fit_{a}+d\cdot Fit_{m}}\\ Fit_{d}=\frac{\sum D_{segment,i}}{\sqrt{{\left(x_{end}-x_{start}\right)}^{2}+{\left(y_{end}-y_{start}\right)}^{2}}}\\ Fit_{T}=\frac{\sum \Delta T_{segment,i}}{N-1}\\ Fit_{mal}=\frac{\sum_{i=1}^{N}\left|y_{i}\right|}{N}\\ Fit_{ang}=\frac{\sum_{i=1}^{N}\left(\pi -\theta \right)}{\pi } \end{array}\right.$$

where $Fit_{d}$, $Fit_{T}$, $Fit_{mal}$, and $Fit_{ang}$ denote the evaluation functions for distance, time cost, malposition, and turning angle, respectively. And $G_{acc}$, $G_{vel}$, and $G_{col}$ present the hard kinetic evaluation index of acceleration, velocity, and collision avoidance. *a*,*b*,*c*,*d* are parameters set by the researcher.

The fitness function mechanism is the key to ensuring good convergence and screening mechanism in the genetic algorithm. We restricted many variables relating to dynamics in the fitness function. To control the motion attitude and smoothness of the vehicle, we optimized the acceleration of each route segment and screened the randomly generated waypoint velocities.

Due to the fact that all waypoint information is packaged through the communication system to the USV end, which is controlled and processed by controllers to optimize path details and tracking effects, optimizing variables such as turns and malposition in path planning algorithms can not only reduce the computational burden of controllers such as LOS, but also facilitate the effective control of the mechanical motion of the propeller by controllers such as PID. The USV’s motion is based on controlling the left and right propellers, and smooth propeller motion is conducive to dealing with ocean current disturbances.

### 3.3. Stable Navigation Trajectory Tracking Control for Stern Ramp Recovery

The trajectory tracking algorithm calculates the heading angle and velocity of the USV based on its current position, velocity state, and desired trajectory curve. This algorithm guides the USV to continuously approach the target trajectory, similar to missile guidance principles. Notable guidance laws for trajectory tracking include LOS, Follow the Carrot (FTC), Constant Bearing (CB), and Proportional Navigation (PN). Among these, the LOS algorithm stands out for its ease of use, reliability, and robustness, which have been extensively validated through research investigations [37]. Therefore, this study focuses on investigating the trajectory tracking algorithm using the LOS method.

**Figure 9:**
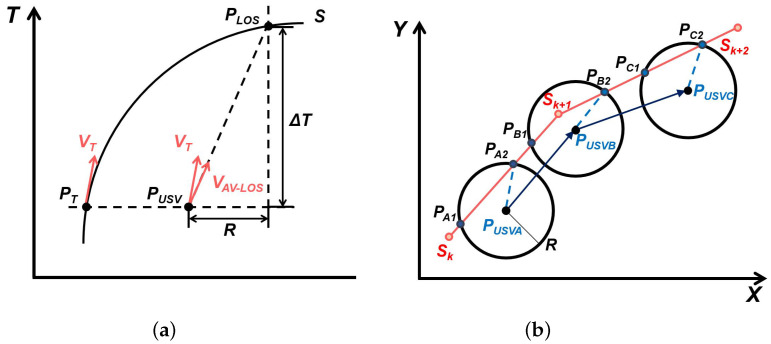
(**a**) The theory of the traditional los. (**b**) The theory of Average Velocity Line of Sight (AV-LOS).

As shown in Figure 9a, the classical LOS algorithm operates by determining the target heading for guiding the USV based on the forward viewpoint along the target path. Initially, the forward way distance R is established. This is accomplished by considering the current position of the USV as the circle’s center and setting the radius of the forward way distance R. The intersection point between the circle and the desired path represents the forward viewpoint. Subsequently, the target heading angle is derived from the line connecting the current point to the forward way point. This approach transforms the problem of tracking the desired path into a heading angle control problem. By utilizing the line between the current point and the forward way point, the target heading angle is obtained, thereby resolving the challenge of path tracking through heading angle control. The selection of the forward way distance R significantly impacts the tracking performance. If R is excessively large, rapid correction of track deviations becomes challenging, while an excessively small R may induce track oscillation [38].

The current LOS and its improved algorithms are limited to guidance in the spatial domain only, which means that it can only derive a target heading from the forward viewpoint and has a very limited ability to handle speed. This means that it can only derive a target heading from the forward viewpoint, and has a very limited ability to handle speed. For this purpose, we introduce an enhanced algorithm called Average Velocity Line of Sight (AV-LOS).

(13) $$V_{AV-LOS}=\frac{R}{\Delta T}$$

where ΔT denotes the time lag between the current position and the forward viewpoint.

As shown in Figure 9b, the planned trajectory S is projected on the two-dimensional plane $\left(X,Y,Z\right)$ and contains the path points $S_{K}$, $S_{K+1}$, $S_{K+2}$, and the actual trajectory of the USV passes through the point $P_{USVA}$, $P_{USVB}$, $P_{USVC}$ in turn, and the forward viewpoints determined by the forward view distance *R* are $P_{A1}$, $P_{A2}$, $P_{B1}$, $P_{B2}$, $P_{C1}$, $P_{C2}$, and at the beginning moment, two forward viewpoints $P_{A1}$ and $P_{A2}$ are solved by the forward view circle and the segment $S_{K}-S_{K+1}$. At the beginning moment, two forward viewpoints $P_{A1}$ and $P_{A2}$ are solved from the forward view circle and the line segment $S_{K}-S_{K+1}$, and at this time, $P_{A2}$, which is closer to the point $S_{K+1}$, is selected as the solution forward viewpoint. When the USV moves to $P_{USVB}$, there is only one forward viewpoint on the track segment $S_{K}-S_{K+1}$, and at this time, $P_{B2}$ is obtained by incorporating $S_{K+1}-S_{K+2}$ into the solution and is used as the solution forward viewpoint. When the USV is located in $P_{USVC}$, $S_{K}$ is out of the team and $P_{C2}$ is selected as the solution forward viewpoint. When the unmanned boat is in $P_{USVC}$, $S_{K}$ is out of the group and $P_{C2}$ is chosen as the forward viewpoint of the solution.

In order to enhance the anti-interference capability of the tracking algorithm, we conducted data tests on simulated ocean waves using the aforementioned methods. Wind and wave data for various sea conditions, such as wave height, were obtained from the International Sea State Scale. Since it is not feasible to measure the level of interference caused by waves during actual operation, we could only apply pre-set compensation measures against such interference. Multiple experiments were conducted in the ship pool laboratory, revealing a maximum deviation of 4.94° for the heading angle and 0.16 m for alignment.

After performing calculations based on the formula presented in Section 3.1, the compensated acceleration is incorporated into the AV-LOS algorithm to enhance the anti-interference capability of the recovery system.

## 4. Experiments

In this section, we will present the hardware model of the stern ramp recovery system along with the underlying framework logic. Additionally, we will showcase the outcomes of simulation and field experiments conducted using the platform. The results will be analyzed using quantitative indicators and graphical representations for a comprehensive evaluation.

### 4.1. Experimental Conditions and Hardware System

**Figure 10:**
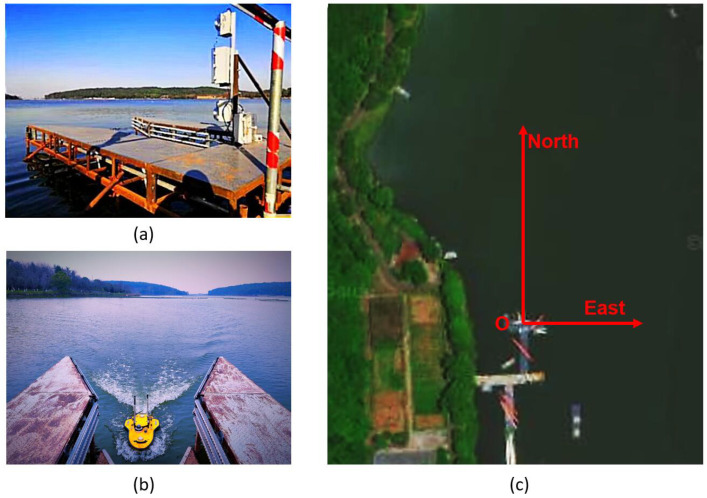
The actual situation of the experimental platform. (**a**) The photo of our platform, which acts as a model of stern ramp. (**b**) The picture records the moment when our USV finishes the recovery process. (**c**) The satellite map of our platform and the directions given are relevant to the coordinate system in the following pictures that we show to describe our experimental results.

At the mother ship’s end, a fixed steel transom chute is installed at the shore to replicate the mother ship for simplicity. The steel transom chute features an upward-sloping U-shaped opening with cylindrical rolling sheaves on the inside, as depicted in Figure 10. The hardware system is constructed on a sturdy fixed steel transom chute structure, which offers ample space and structural stability. Similarly, the hardware system at the mother ship end can be categorized into three sub-modules: computation and control module, sensor module, and power module.

**Figure 11:**
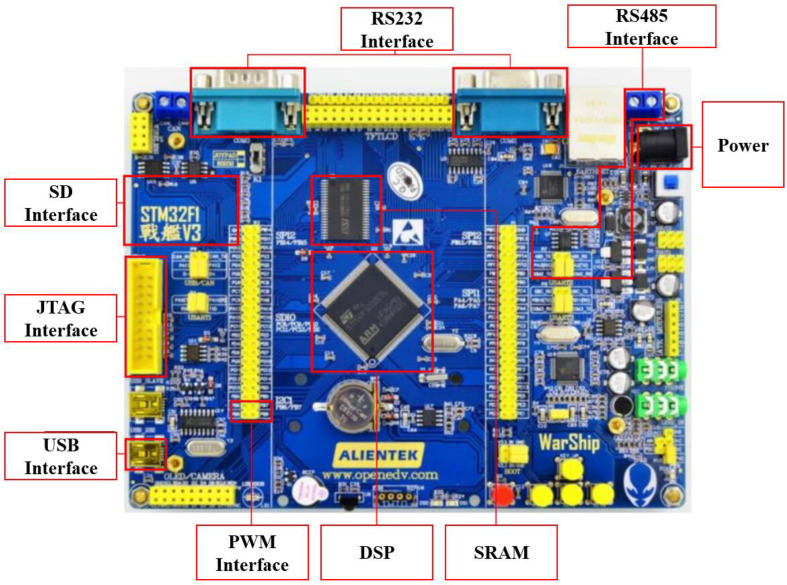
The layout of MCU. Equipment (Guangzhou YuanziDianzi Electronic Technology Co., Ltd, Guangzhou, China) has been modified.

**Figure 12:**
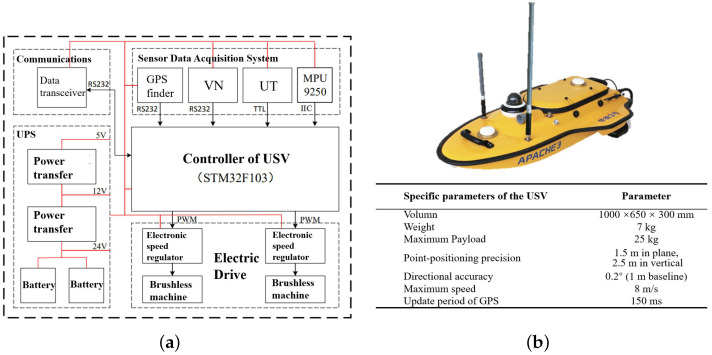
(**a**) The framework of the hardware system of our USV. (**b**) The main parameters of our USV. (Shell from Shanghai Huace Navigation Technology Ltd., Shanghai, China).

As shown in Figure 11, the boat-side controller described in this paper uses the STM32F103ZET6, and the controller has a wealth of interface resources. We assembled the hardware system, where the hardware system was structured as shown in Figure 12a. The USV used in the experiment with its information is shown in Figure 12b.

**Figure 13:**
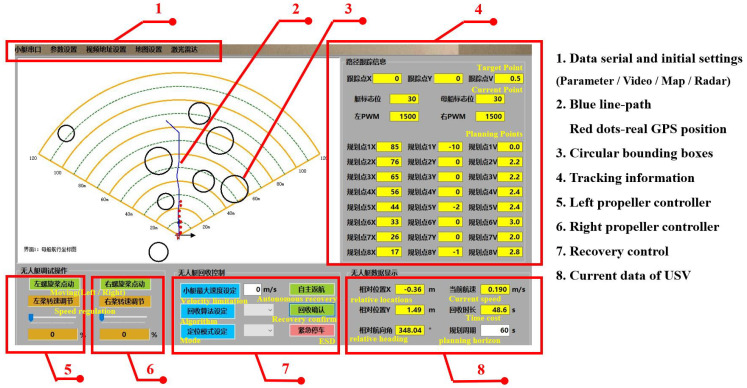
Operation interface of the upper computer.

To streamline the development process, we opted for Visual Studio 2022 (Microsoft, SEA, Redmond, WA, USA) and utilized the C# language to create a Windows application. The upper computer was developed using the C# language, and its software interface is illustrated in Figure 13.

After constructing the hardware/software system for the recovery system as outlined in the preceding section, a series of tests were conducted in lake waters. The primary objective was to validate the fundamental functionalities of the recovery system. Upon successfully debugging the system’s hardware and software components, a dedicated recovery scenario was devised to facilitate a series of recovery tests. These tests aimed to evaluate the effectiveness of the improved genetic algorithm (IGA) path planning algorithm and the AV-LOS tracking algorithm.

### 4.2. The Simulation of IGA and AV-LOS

By distinguishing between the length of the route plan, the size of the corners, and the number of surrounding obstacles, we designed four arithmetic examples with different starting points, as shown in Table 1.

**Figure 14:**
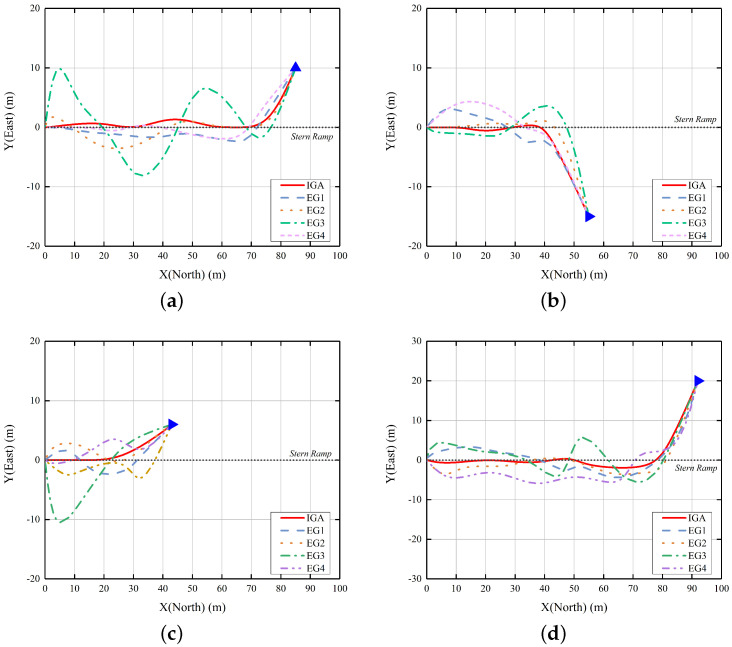
The comparative results of simulation outcomes of four examples. The blue triangle represents the approximate orientation of the starting point towards the USV.(**a**) Case 1; (**b**) Case 2; (**c**) Case 3; (**d**) Case 4.

By incorporating control variables, we have formulated these computational scenarios. The simulation outcomes will be depicted in Figure 14, where EG1 denotes the absence of population initialization enhancement, EG2 signifies the absence of SSIH, EG3 employs a simplistic linear penalty function, and EG4 indicates the lack of an Elitism Strategy.

After generating each set of operating algorithms, we applied curve fitting using β-spline curves to all pathways and conducted a comparative analysis. It is evident that the IGA item displays a relatively strong performance of malposition across all four sets of examples, particularly in terms of proximity to the end of the central axis of the stern ramp, denoted by (0, 0).

Moreover, the deficiency of a multidimensional evaluation system has contributed to the notably poor performance of the EG3 item across all four algorithms. It is apparent that there are numerous challenging corners in practical implementation, resulting in compromised path smoothness. In conjunction with the previously mentioned evaluation metrics formulas, we conducted a comprehensive comparison of the evaluation metric values for malposition and large angles in the IGA and EG3 samples across the four examples. The results have been carefully tabulated and are presented in Table 2. Based on the table, it is evident that the smoothing metrics of the IGA and EG3 in all cases exhibit a significant difference of approximately two-fold. This observation suggests that the unimproved fitness function lacks efficacy in terms of path smoothing selection.

This is because the convergence and screening mechanism of the genetic algorithm is closely related to the design of the fitness function. The fitness function of IGA strictly limits the recovery attitude requirements for stern ramp recovery to moderate and heading. In addition, EG3 also lacks constraints on the path smoothness parameter indicators, resulting in uneven speed and frequent sharp turns.

**Figure 15:**
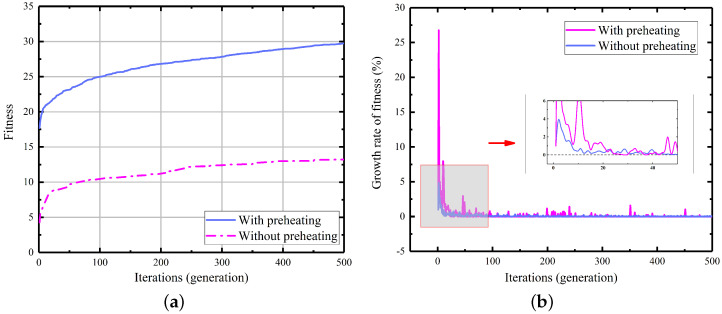
(**a**) The actual fitness function values. (**b**) The growth rate of fitness.

To validate the effectiveness of the preheating process in improving the genotype quality of the initial population and consequently reducing the number of iterative generations required, we conducted 100 experiments utilizing an arithmetic case sample with a starting point of (85, 10). These experiments were carried out both with and without the preheating operation. We set the number of elite groups for the experiments to 10. Moreover, to ensure better visual interpretation of the results, we enforced a fixed number of iterations per dataset at 500, which significantly exceeds the number of iterations typically needed for path planning. By recording the average fitness function values for the elite group in each generation, we analyzed and presented the results in Figure 15.

Based on the experimental results, it is evident that the example utilizing the preheating process exhibits a higher fitness function value in the initial stage of the algorithm, indicating its effectiveness in improving the genotype quality. As depicted in Figure 15, although the algorithm without the preheating process demonstrates a higher growth rate of fitness values, the fitness function value of the offspring progeny is still constrained by the parent population. This indicates that the parent genes that underwent population initialization optimization have good phenotypes. Conversely, the algorithm incorporating the preheating process tends to reach a saturation point in its growth rate within approximately 20 generations. In our extensive simulations, we have observed that this is indeed the number of iterations required for the IGA to successfully accomplish path planning.

The Elite Strategy and SSIH contribute to an accelerated convergence rate primarily by enhancing genetic manipulation techniques, as evidenced by the time taken for computational planning displayed in Table 3. Additionally, they exhibit some positive impact on path smoothness. Table 4 presents the comparative metrics illustrating these effects.

It is evident that the incorporation of the Elite Strategy or SSIH yields advantages in enhancing the convergence rate. As the planning difficulty increases, there is a notable increase in computation time. However, these methods operate through distinct mechanisms. The former safeguards the retention of high-quality individuals during the genetic process by preserving their genotypes in each generation. On the other hand, the latter facilitates genetic diversity by releasing redundant memory and generating novel samples.

Based on the comparison of dimensionless evaluation metrics, it is observed that the planning results achieved by IGA exhibit superior performance in terms of smoothness, path distance, and time consumption compared to the other two algorithms. However, it should be noted that there are instances where the differences in metrics for some data are not particularly significant. To improve the overall quality of the planned path, our fitness function adopts a weighting strategy. Even if the fitness function values of the final screening are significantly different, some qualitative indicators may be similar.

The AV-LOS algorithm simulation is implemented using MATLAB. As an illustrative example, the desired trajectory S in (X, Y, T) space is composed of three motion segments concatenated together. The segments consist of $P_{1}$(0, 0, 0), $P_{2}$(0, 20, 8), $P_{3}$(30, 20, 14), and $P_{4}$(30, 0, 22). These segments collectively form an inverted ’U’ shape in the X/Y plane, effectively simulating tracking scenarios involving acceleration, deceleration, and right-angle turns typical for unmanned boats. The simulation control period is denoted as T = 0.2, and the algorithm utilizes a forward-looking distance of *R* = 3, and the following assumptions are made:

- The USV utilizes a fixed control period T for speed and heading control. At the start of each period, the desired heading and desired speed are determined by solving the guidance law.
- The hysteresis effect in heading control is disregarded, and changes in speed are treated as linear transformations that either increase or decrease over time.

**Figure 16:**
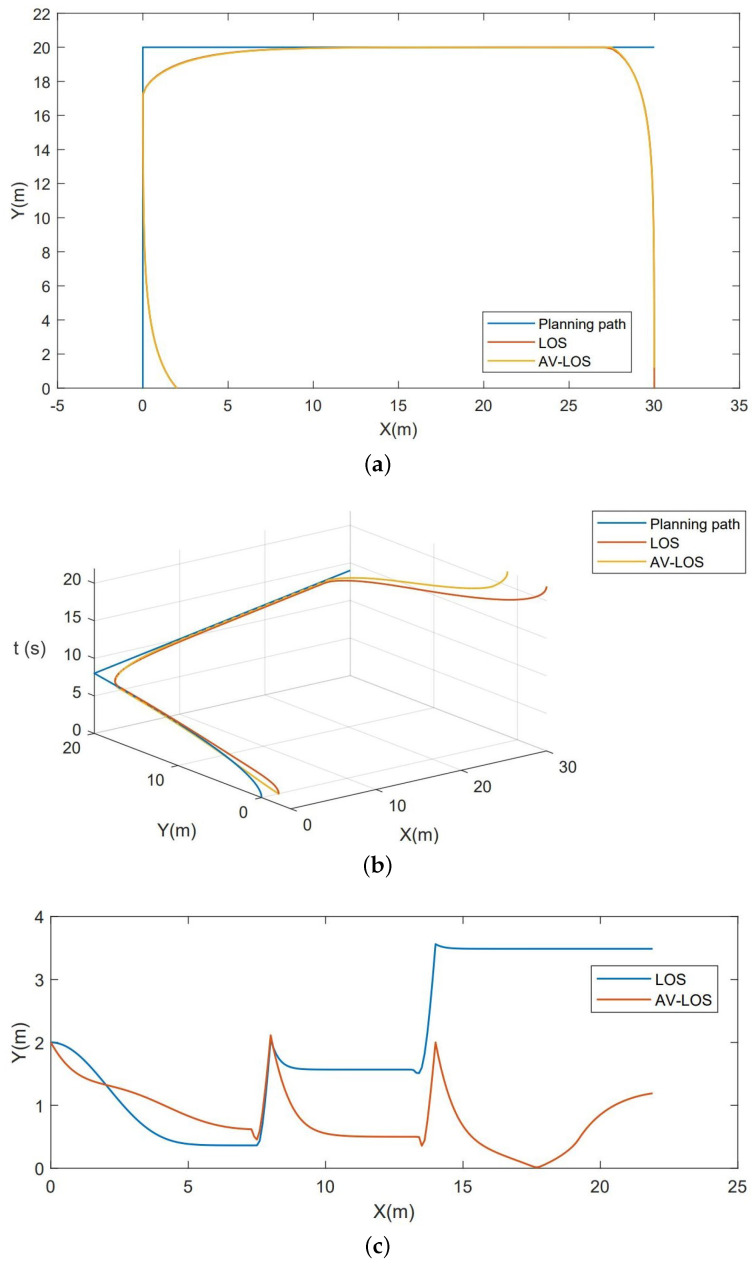
The simulation result of AV-LOS. (**a**) Two-dimensional projection for trajectory tracking. (**b**) Three-dimensional projection for trajectory tracking. (**c**) Tracking simulation with starting point (2, 0, 0).

The simulation results are shown in Figure 16.

The formula for trajectory deviation B(t) can be expressed as follows:

(14) $$B\left(t\right)=\sqrt{{\left(X_{t}\left(t\right)-X_{r}\left(t\right)\right)}^{2}+{\left(Y_{t}\left(t\right)-Y_{r}\left(t\right)\right)}^{2}}$$

where $X_{t}\left(t\right)$ and $Y_{t}\left(t\right)$ denote the coordinates of the desired trajectory and $X_{r}\left(t\right)$ and $Y_{r}\left(t\right)$ represent the coordinates of the real trajectory.

It is evident that the deviation of the 2D trajectory is minimal. Nonetheless, owing to the inclusion of the forward view distance, the actual trajectory undergoes smooth transitions with certain curvature during right-angle turns. Consequently, a deviation emerges at the termination point of the 3D trajectory. The LOS trajectory deviation takes the form of a three-step staircase since it directly adopts the isochronous target speed as the desired speed. Employing a random function, generate 50 sets of initial coordinates and repeat the simulation 10 times for each set. Calculating the average trajectory deviation for each algorithm, the resulting average deviation for the LOS algorithm is determined to be 2.0402 m, whereas, for the AV-LOS algorithm, it is found to be 0.8161 m.

### 4.3. The Field Tests of IGA Incorporating AV-LOS

**Figure 17:**
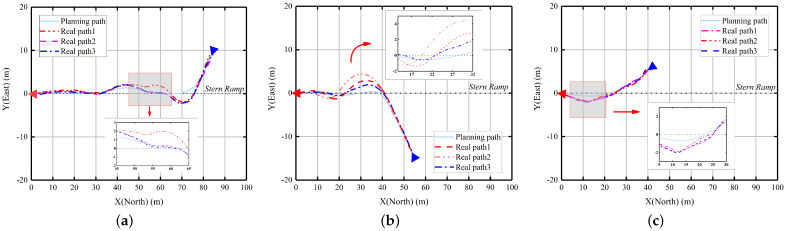
The result of field tests using IGA incorporating AV-LOS. (**a**) Case 1; (**b**) Case 2; (**c**) Case 3.

We selected the initial three samples, which demonstrate significant variations in their planning characteristics, for conducting field experiments. In order to investigate the trajectory tracking performance of AV-LOS under various wind and wave interference scenarios, we recorded the first set of field-tested waypoint planning data and replicated the experiment twice at different times. The resulting actual paths of each experiment are denoted as Real path1, Real path2, and Real path3, respectively. The real GPS coordinates obtained from these experiments are visualized using a β-spline plot, as depicted in Figure 17.

**Figure 18:**
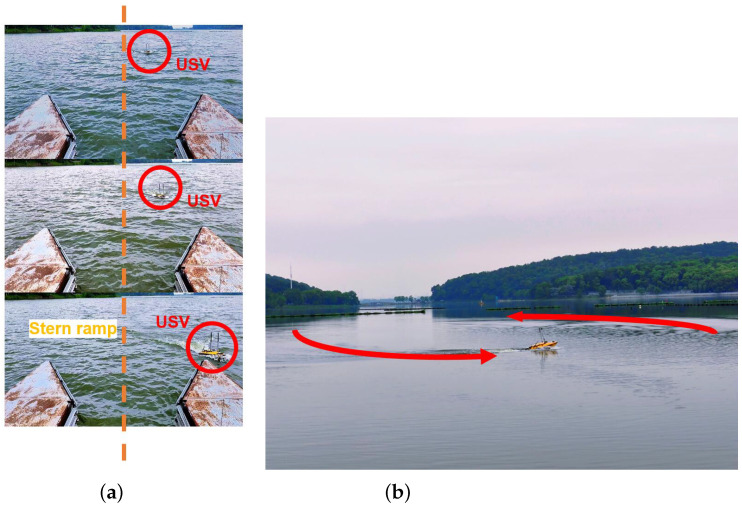
(**a**) The example when the USV is unable to meet the demand for malposition because the tracking radius *R* is too big. After the position in the third picture, the USV will hit the steel plate when heading to the next waypoint. (**b**) When the tracking radius *R* is too small, the USV made a turn at the current waypoint.

It is evident that the actual planning results closely resemble the simulation results, with a slight deviation observed during larger corners. This discrepancy can be attributed to varying disturbance conditions, which result in different magnitudes of translation for the USV during the cornering process. This phenomenon is closely associated with the tracking radius R set in the AV-LOS algorithm. As depicted in Figure 18, setting a tracking radius R that is too large causes an arc offset in the waypoints during tracking, while setting it too small hinders the upper computer’s ability to assist the USV in tracking the next waypoint, leading to the USV making a turn with the current waypoint as the center.

Additionally, it is observed that the actual trajectory tends to have larger corners compared to the planning path. This is due to the necessity for the USV to undergo a dynamics adjustment process while making turns in a water environment. However, it is crucial for path planning to avoid sharp corners, as they can render the waypoint tracking ineffective during the dynamics adjustment process and result in a significant deviation from the intended course. Although the simulation results demonstrate that AV-LOS exhibits a small tracking error, a strategy solely focused on speed-oriented tracking is not an optimal solution for this issue.

**Figure 19:**
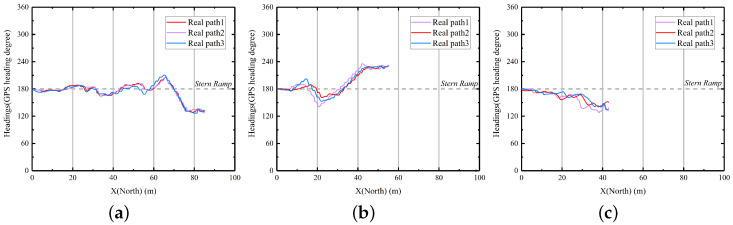
The result of field tests using IGA incorporating AV-LOS. (**a**) Case 1; (**b**) Case 2; (**c**) Case 3.

**Figure 20:**
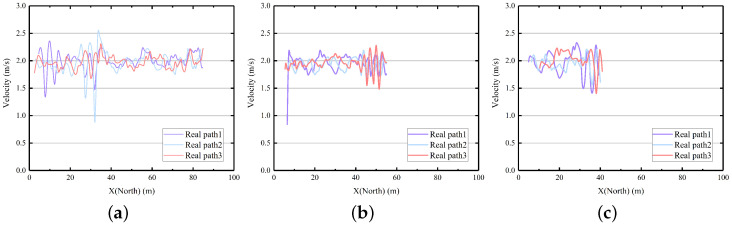
The result of field tests using IGA incorporating AV-LOS. (**a**) Case 1; (**b**) Case 2; (**c**) Case 3.

To provide a more comprehensive analysis of the system’s actual planning effectiveness, we conducted a thorough examination of the collected heading and speed data, as shown in Figure 19 and Figure 20.

From Figure 19, we can see that the heading of the USV in water tends to be basically stable without significant fluctuations. Due to the influence of water flow, there are slight differences in the headings of the three path instances. It can be observed that as the x coordinate decreases, the heading gradually converges to 180°. This is due to the alignment of the USV to the centerline of the stern ramp during the recovery process and preparation for pitch correction. The difference between the heading angle and 180° does not exceed 60° in any of the examples, indicating that there were few sharp turns.

We removed the process of rapid speed changes during the start and end phases and analyzed the speed changes of the USV during the driving phase. It can be observed from Figure 20 that when the planning start point is far from the target, the planning path of the USV is longer and the speed uniformity is better. However, when the planning path is short and the longitudinal distance between the start and end points is large, the speed stability is not as good as the former. However, the difference between the maximum speed and the minimum speed usually does not exceed 1 m/s, which indicates that the anti-interference ability of the AV-LOS algorithm has a certain effect. This also indicates that there are still many areas for improvement in our work in the future.

## 5. Discussion

This paper presents an improved genetic algorithm for a stern ramp recovery system of the USV. The genetic algorithm, known for its global planning capabilities and retrieval efficiency, has proven to be highly practical. However, traditional two-dimensional modeling approaches and single fitness evaluation systems significantly increase the time required for path planning.

In the realm of algorithm optimization, we validated the viability of a genetic algorithm utilizing 3D modeling alongside a novel coding mechanism. By enhancing genetic operations to boost progeny diversity, we achieved a substantial improvement in the convergence rate. Furthermore, the multifactor screening of the fitness function enhances efficiency, albeit placing higher demands on the formulation of linear coefficients. By incorporating the forces exerted on the USV in real water environments through accurate modeling, we have integrated them into the enhanced AV-LOS algorithm. Furthermore, a method diagram for the AV-LOS algorithm is provided. Through simulation experiments, it has been demonstrated that the AV-LOS algorithm outperforms the traditional LOS algorithm in terms of tracking performance.

We have built a USV experimental platform and designed a hardware system. In practice, the optimized genetic algorithm demonstrates adaptability when dealing with longer distances that involve larger detours. However, due to surface disturbances, the actual path generated using the IGA might experience partial shifts, potentially resulting in less favorable detours. Nonetheless, it possesses an advantage in terms of malposition and being able to return to the original route promptly with the assistance of AV-LOS.

However, our system still has several shortcomings. One is that the movement of the obstacles set up in the experiment follows a regular pattern, which may not fully reflect real-world scenarios. The second is that the attitude adjustment of the USV during navigation may occasionally widen the turning path segment, even though the disturbance of the water surface environment is taken into account. And finally, when planning paths with larger turns and smaller total lengths, minor speed fluctuations may occur. In future efforts, we aim to further optimize the hardware system and the structure of the algorithm, while proposing reasonable solutions to deal with these limitations. 

## Figures and Tables

**Table 1:** Specific information of planning examples for recovery.

Case	Start Point	End Point
1	(85, 10)	(0, 0)
2	(55, −15)	(0, 0)
3	(43, 6)	(0, 0)
4	(92, 20)	(0, 0)

**Table 2:** Scalar analysis of smoothing indicators for modified fitness functions.

Case	$F{\mathrm{it}}_{\mathrm{ang},\mathrm{IGA}}$	$F{\mathrm{it}}_{\mathrm{ang},\mathrm{EG}3}$	$F{\mathrm{it}}_{\mathrm{mal},\mathrm{IGA}}$	$F{\mathrm{it}}_{\mathrm{mal},\mathrm{EG}3}$
1	0.48	1.22	1.30	2.40
2	0.42	0.86	2.57	3.86
3	0.37	0.69	2.17	3.00
4	1.44	2.75	4.55	5.82

**Table 3:** Comparison of the time consumption for iterations.

Case	${\mathrm{Time}}_{\mathrm{IGA}}$	${\mathrm{Time}}_{\mathrm{EG}2}$	${\mathrm{Time}}_{\mathrm{EG}4}$
1	2.067	11.392	12.769
2	2.683	8.430	8.319
3	1.556	7.436	6.366
4	3.792	15.985	62.00

**Table 4:** Comparison of evaluation metrics for the planning results of Improved Genetic Algorithm (IGA), EG2, and EG4 algorithms.

Case	$\mathrm{Fi}t_{\mathrm{ang},\mathrm{IGA}}$	$\mathrm{Fi}t_{\mathrm{ang},\mathrm{EG}2}$	$\mathrm{Fi}t_{\mathrm{ang},\mathrm{EG}4}$	$\mathrm{Fi}t_{\mathrm{mal},\mathrm{IGA}}$	$\mathrm{Fi}t_{\mathrm{mal},\mathrm{EG}2}$	$\mathrm{Fi}t_{\mathrm{mal},\mathrm{EG}4}$	$\mathrm{Fi}t_{d,\mathrm{IGA}}$	$\mathrm{Fi}t_{d,\mathrm{EG}2}$	$\mathrm{Fi}t_{d,\mathrm{EG}4}$	$\mathrm{Fi}t_{T,\mathrm{IGA}}$	$\mathrm{Fi}t_{T,\mathrm{EG}2}$	$\mathrm{Fi}t_{T,\mathrm{EG}4}$
1	0.48	0.59	0.60	1.30	2.00	2.00	1.05	1.14	1.15	5.18	5.40	5.34
2	0.42	0.83	0.63	2.57	3.14	2.71	1.08	1.13	1.15	5.17	5.68	4.80
3	0.37	0.76	0.75	2.17	2.17	2.33	1.02	1.11	1.09	4.95	5.60	5.60
4	1.44	1.44	3.44	4.55	4.55	3.73	1.19	1.19	1.17	5.09	5.00	5.74

## Data Availability

Data sharing not applicable.

## References

[1] Arzamendia M., Espartza I., Reina D.G., Toral S., Gregor D. (2019). Comparison of eulerian and hamiltonian circuits for evolutionary-based path planning of an autonomous surface vehicle for monitoring ypacarai lake. J. Ambient. Intell. Humaniz. Comput..

[2] Peng Z., Wang J., Wang D., Han Q.L. (2020). An overview of recent advances in coordinated control of multiple autonomous surface vehicles. IEEE Trans. Ind. Inform..

[3] Song R., Liu Y., Bucknall R. (2019). Smoothed A* algorithm for practical unmanned surface vehicle path planning. Appl. Ocean. Res..

[4] Liu Z., Zhang Y., Yu X., Yuan C. (2016). Unmanned surface vehicles: An overview of developments and challenges. Annu. Rev. Control..

[5] Weihai Skysailing Technology (2021). SKYSAILING. http://tianfanzhineng.com/product/info/1/.

[6] SUITOR (2023). Unmanned System. https://www.4008075595.com/content_2_12_122.html.

[7] Ku Y.N.Z. (2022). Analysis of the Development of USV’s Technology and Operational Styles Abroad. https://www.eet-china.com/mp/a158815.html.

[8] Carboniferous USVs Launched to Assist in Water Environmental Protection. https://stjep.cn/article.

[9] China Economic Net (2014). Israel Unveils Unmanned Combat Marine System. http://en.ce.cn/World/Middleeast.

[10] Observer S. (2022). Türkiye Has Developed the “Marlin” Unmanned Boat, and the Era of Unmanned Combat at Sea is Coming?. https://export.shobserver.com/baijiahao/html/538455.html.

[11] Xie Y. (2017). China’s First Comprehensive Geological Survey of Coastal Zones Using Unmanned Boats. https://www.shu.edu.cn/info/1055/58977.htm.

[12] Yunzhou Intelligence (2019). Alumni Enterprise Yunzhou Intelligent Will Participate in the Tongji University Alumni Industry Expo. https://www.163.com/dy/article/ESNP6V960518SJIU.html.

[13] Eads M.K.E. (2017). Well Deck Entry. https://www.defense.gov/Multimedia/Photos/igphoto/2002038005/.

[14] Chuang G.Z. (2019). The Invention Patent for the Stern Slide of Guangzhou Shipyard Won the first Chinese Patent Silver Award. https://www.sohu.com/picture/286333857.

[15] Sohu (2022). What Kind of Wonderful Sights Can You See If You Dive into the 7000-Meter-Deep Sea?. http://it.sohu.com/a/576939130_121118997.

[16] Singh Y., Sharma S., Sutton R., Hatton D., Khan A. (2018). A constrained A* approach towards optimal path planning for an unmanned surface vehicle in a maritime environment containing dynamic obstacles and ocean currents. Ocean. Eng..

[17] Kim Y.H., Son W.S., Park J.B., Yoon T.S. (2016). Smooth path planning by fusion of artificial potential field method and collision cone approach. MATEC Web Conf..

[18] Sang H., You Y., Sun X., Zhou Y., Liu F. (2021). The hybrid path planning algorithm based on improved A* and artificial potential field for unmanned surface vehicle formations. Ocean. Eng..

[19] Lee H.C., Yaniss T., Lee B.H. (2012). Grafting: A path replanning technique for rapidly-exploring random trees in dynamic environments. Adv. Robot..

[20] Khan A.H., Li S., Luo X. (2019). Obstacle avoidance and tracking control of redundant robotic manipulator: An RNN-based metaheuristic approach. IEEE Trans. Ind. Inform..

[21] Wang N., Xu H., Li C., Yin J. (2021). Hierarchical path planning of unmanned surface vehicles: A fuzzy artificial potential field approach. Int. J. Fuzzy Syst..

[22] Xin J., Zhong J., Yang F., Cui Y., Sheng J. (2019). An improved genetic algorithm for path-planning of unmanned surface vehicle. Sensors.

[23] Pettersen K.Y., Lefeber E. Way-point tracking control of ships. Proceedings of the 40th IEEE Conference on Decision and Control (Cat. No. 01CH37228).

[24] Fossen T.I., Pettersen K.Y. (2014). On uniform semiglobal exponential stability (USGES) of proportional line-of-sight guidance laws. Automatica.

[25] Caharija W., Pettersen K.Y., Sørensen A.J., Candeloro M., Gravdahl J.T. (2014). Relative velocity control and integral line of sight for path following of autonomous surface vessels: Merging intuition with theory. Proc. Inst. Mech. Eng. Part M J. Eng. Marit. Environ..

[26] Fossen T.I., Pettersen K.Y., Galeazzi R. (2014). Line-of-sight path following for dubins paths with adaptive sideslip compensation of drift forces. IEEE Trans. Control. Syst. Technol..

[27] Liu L., Wang D., Peng Z. Predictor-based line-of-sight guidance law for path following of underactuated marine surface vessels. Proceedings of the 2015 Sixth International Conference on Intelligent Control and Information Processing (ICICIP).

[28] Wu N., Chen H. (2013). Simulation design on dynamic positioning system of vessels. Ship Sci. Technol..

[29] Song J., Du J., Li W., Sun Y., Chen H. (2011). Simulation of ship dynamic positioning system disturbance due to wave. J. Dalian Marit. Univ..

[30] Zhuang J., Su Y., Liao Y., Sun H. (2012). Unmanned Surface Vehicle Local Path Planning Based on Marine Radar. J. Shanghai Jiao Tong Univ..

[31] Zhu C., Liang X. (2008). Novel genetic algorithm with multi-elitist preservation method. J. Comput. Appl..

[32] Ma D. (2018). Unmanned Surface Vehicle Path Planning Based on Immune Genetic Algorithm. Master’s Thesis.

[33] de Jong Kenneth A. (1975). Analysis of the Behavior of a Class of Genetic Adaptive Systems.

[34] Zarándy Á., Nemeth M., Nagy Z., Kiss A., Santha L., Zsedrovits T. (2016). A real-time multi-camera vision system for UAV collision warning and navigation. J. Real-Time Image Process..

[35] Deb K. (2000). An efficient constraint handling method for genetic algorithms. Comput. Methods Appl. Mech. Eng..

[36] Khan A.H., Li S., Chen D., Liao L. (2020). Tracking control of redundant mobile manipulator: An RNN based metaheuristic approach. Neurocomputing.

[37] Breivik M., Fossen T.I. (2007). Applying missile guidance concepts to motion control of marine craft. IFAC Proc. Vol..

[38] You S. (2022). A Dissertation Submitted in Partial Fulfillment of the Requirements for the Master Degree in Engineering. Master’s Thesis.

